# Pachydermoperiostosis: a case report of initial improvement with etoricoxib

**DOI:** 10.1097/MS9.0000000000001146

**Published:** 2023-08-14

**Authors:** Nirish Vaidya, Nabaraj Acharya, Shreesuna Katila, Samyog Adhikari, Urmila Pandey

**Affiliations:** a Departments of Internal Medicine and; b Orthopedics and Traumatology, Kathmandu University School of Medical Sciences, Dhulikhel Hospital, Dhulikhel, Nepal

**Keywords:** case report, etoricoxib, hypertrophic osteoarthropathy, Nepal, pachydermoperiostosis

## Abstract

**Introduction and importance:**

Pachydermoperiostosis (PDP) is a syndrome characterised by the triad of pachydermia, digital clubbing and periostosis of long bones and its scarce incidence and similarity in clinical features with acromegaly makes the diagnosis challenging. The elevated PGE2 levels have been hypothesised as one of its mechanisms and therapies have been targeted to inhibit this prostaglandin.

**Case presentation:**

A 25-year-old man with no comorbidities presented to OPD with a 10-year history of bilateral pain and swelling of the hands and feets associated with hyperhidrosis, grade IV clubbing and marked skin thickening on his forehead. X-rays revealed hyperostosis of the metacarpals, proximal and middle phalanges and periosteal bone formation with cortical thickening of the ankle joint. Tests done to rule out differentials such as thyroid acropachy, acromegaly, psoriatic arthritis were normal and a clinical diagnosis of PDP, a rare genetic disease characterised by pachyderma, digital clubbing and periostosis was made.

**Clinical discussion:**

The patient was managed conservatively with etoricoxib for 6 months on a follow-up basis. The symptoms were improving and a repeat X-ray showed partial improvement of soft tissue thickening and periostosis.

**Conclusion:**

PDP is a rare diagnosis with no clear consensus on a management approach. Its management with selective COX-2 inhibitors such as etoricoxib should be considered but its long-term effects should be studied further.

## Introduction

Highlights

- Pachydermoperiostosis is a rare diagnosis.
- Due to its rarity, therapeutic management remains a challenge.
- Management with etoricoxib has shown promising results.
- The long-term use of etoricoxib should be studied further.

Pachydermoperiostosis (PDP) is a rare disorder characterised by the triad of pachydermia (thickening of skin), digital clubbing (acropachy) and periostosis of long bones^1^. PDP also known as Touraine-Solente-Gole syndrome is a familial disorder having three forms: complete (periostosis and pachyderma), incomplete (without pachyderma) and the forme fruste (pachydermia with minimal skeletal changes)^2^.

PDP has 0.16% prevalence rate and shows male to female ratio of 7:1, predominantly affecting adolescent males^3,4^. Both autosomal dominant and autosomal recessive forms are existent with variation in clinical severity, intrafamilial variability and prevalence of some features^5^. As the scarce incidence and similarity in clinical features with acromegaly such as acral enlargement and facial appearance changes along with joint pain and excessive sweating makes the diagnosis challenging, the diagnosis of PDP should be established if at least two among family history, clubbing, hypertrophic skin changes and bone pain/radiographic changes are present^4,6^. As most of the cases are diagnosed clinically by ruling out the differentials, inappropriate treatment or late treatment may lead to chronic debilitating complications like severe kyphosis, restricted motion and neurologic manifestations^4^. Elevated PGE2 levels are hypothesised to eventually lead to hyperhidrosis, acro-osteolysis, periostosis, arthritis and pachyderma as seen in PDP patients and the management can be done symptomatically using NSAIDs, corticosteroids, or colchicine^7^. However, steroids and colchicine not being so effective for arthralgia that is thought to be the result of periosteum inflammation, NSAIDs are usually prescribed considering PGE2 as an important pathogenic factor^7^.

Here, after several investigations to rule out other differential diagnoses, a case of a complete form of PDP with severe pain and cylindrical enlargement of the bilateral upper and lower limbs is presented who was treated with etoricoxib for 7 months and showed clinical improvement in symptoms. This case report adds more evidence to the existing articles of etoricoxib treatment in case of hypertrophic osteoarthropathy where one report stated about improvement within 1 week of etoricoxib treatment^8^. This case report has been reported in accordance with the Surgical CAse REport (SCARE) criteria^9^.

### Case presentation

A 25-year-old man residing in the hilly district of Nepal presented with a 10-year history of pain and swelling in both hands and feet of insidious onset, progressive in nature without radiation but exacerbated during long travel and dangling of limbs associated with profuse sweating (hyperhidrosis) and progressive enlargement of hands and feet. He also gives a history of easy fatigability, heat intolerance and skin changes like acne, scalp dandruff and thickened eyelids. He had visited multiple health care centres for this concern for the past 10 years as the suspected diagnosis could not be confirmed by any laboratory results. None of the family members had a similar history.

**Figure 1:**
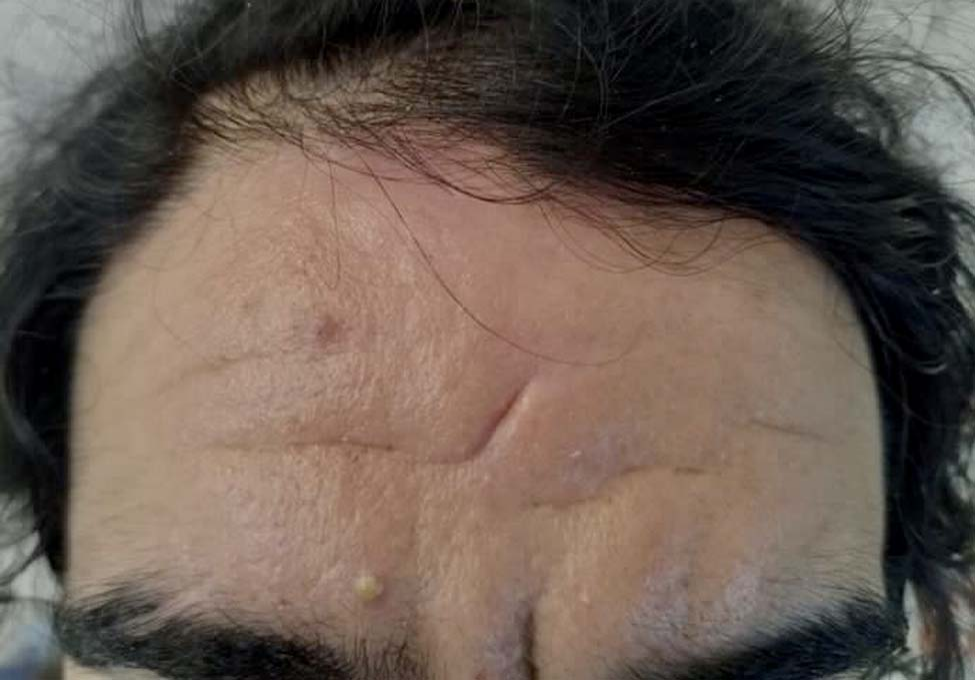
Pachyderma.

**Figure 2:**
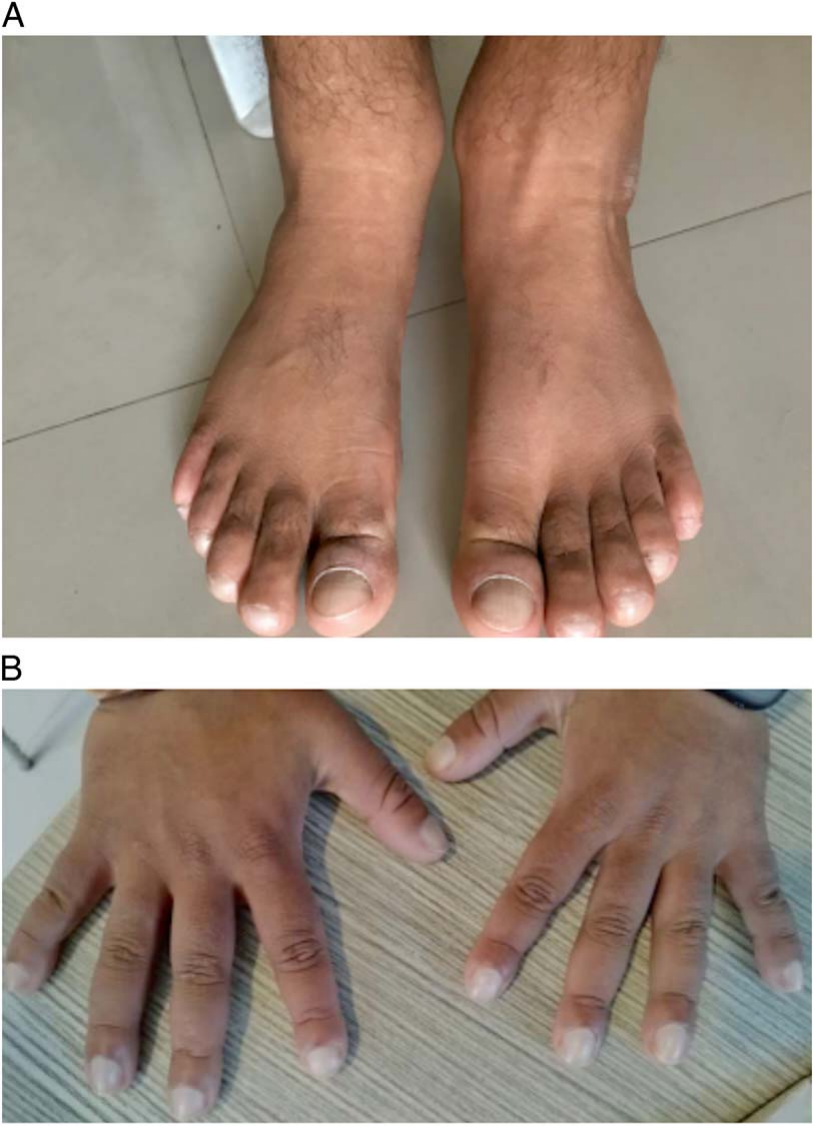
Bilateral soft tissue and joint swelling of (A) Lower limbs and (B) Upper limbs with grade IV clubbing.

The patient was moderately built with marked thickening of skin folds in his forehead (Fig. 1) and evident swelling of bilateral ankle joints along with grade IV clubbing on both upper and lower limbs (Fig. 2). Systemic examinations were otherwise normal. A clinical diagnosis of PDP, a rare genetic disease characterised by pachyderma, digital clubbing and periostosis was made. Normal serum insulin-like growth factor-1 (IGF-1) level and thyroid function tests ruled out our other differentials such as acromegaly and hyperthyroidism. Routine blood investigations including blood counts, liver function tests and renal function tests were also normal. Psoriatic arthritis was ruled out on the basis of the clinical history, which is generally limited to the extremities with psoriatic nail involvement and tests for rheumatoid factor and anti-cyclic citrullinated peptide were normal, ruling out suspicion of rheumatoid arthritis.

**Figure 3:**
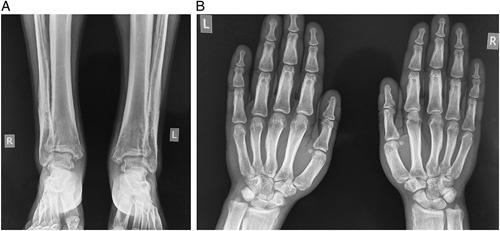
(A) Radiograph of ankle joint showing periosteal bone formation and cortical thickening. (B) Radiograph of bilateral hands showing hyperostosis of metacarpals, proximal, and middle phalanges.

Radiography of the ankle joint showed symmetric, shaggy subperiosteal new bone formation and cortical thickening leading to the characteristic dripping candle wax appearance of the fibula; coarse periosteal new bone formation in the distal tibia and fibula leading to distortion of the medial malleolus and periarticular osteopenia with preserved articular surface along with hyperostosis of the metatarsal bones (Fig. 3).

**Figure 4:**
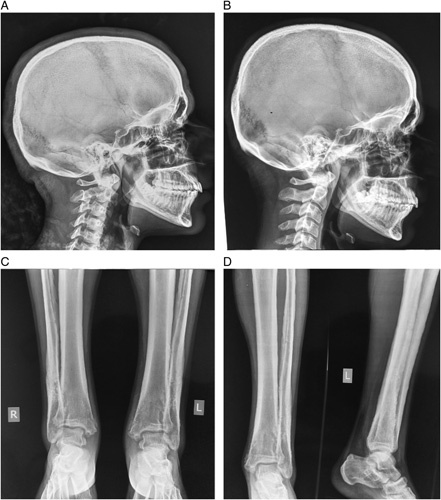
Radiograph showing (A) hyperostosis of skull bones and (B) Improvement in soft tissue thickening after etoricoxib treatment. (C and D) improvement in soft tissue swelling of bilateral ankle joint after 7 months of etoricoxib treatment.

Hyperostosis of the metacarpal bones, proximal and middle phalanx with unaffected articular surfaces along with increased soft tissue shadow showing characteristic sausage finger appearance was also seen in the radiograph, which was supported by the clinical picture (Fig. 3). Hair on the end appearance of the parietal bone along with hyperostosis of the skull bones and normal appearing Sella turcica was witnessed (Fig. 4).

After the diagnosis of PDP was made, he was prescribed with selective COX-2 inhibitor (etoricoxib 90 mg oral once a day) and he showed partial improvement in joint pain and swelling along with a gradual reduction of pachyderma making his daily life easier with treatment thereafter. Additionally, retinoid ointment was also used during this course, which might have improved symptoms of facial acne and skin changes whereas physical therapy also helped to improve joint mobility. Radiological follow-up after 6 months showed improvement in soft tissue thickening near the occiput and supraorbital ridge of the skull (Fig. 4) as well as in soft tissue swelling of the lower limb (Fig. 4).

Continuous pain and change in his physical appearance affected his personal and social life. Ambiguous diagnosis and deteriorating symptoms made the process of seeking medical help overwhelming. Over the course of treatment, the physical and mental well-being is now improving. For follow-up, he visits our outpatient clinic or communicates via telephone with the consultant. The patient experienced no complications from the condition or the medications.

## Discussion

PDP accounts for only 3–5% cases of hypertrophic osteoarthropathy, being a rare differential-diagnosis of chronic bone and joint pain^10^. In the context of Nepal, despite having typical clinical features and radiologic findings, the diagnosis is frequently missed due to the rarity of the disease, poor knowledge, limited resources and resemblance with other conditions. Here the complete form of PDP in a 25-year-old is presented and the analysis of clinical, laboratory and radiological characteristics pointed towards the diagnosis of PDP. These features and the relevant differential diagnoses, taken in consideration form the main points of discussion.

A study of various cases of PDP showed the role of epidermal growth factor, vascular endothelial growth factor and fibroblasts activity in pathogenesis^11,12^. Previous studies have shown that VEGF causes vascular hyperplasia, osteogenesis and oedema thus explaining the classic presentations of HOA^12^. An altered nuclear steroid receptor: epidermal growth factor ratio causes hypertrophy of cutaneous tissues as per some articles^11^. More recent studies suggest prostaglandin mediated pathways as the major contributor of PDP pathogenesis where homozygous mutations in 15-hydroxyprostaglandin dehydrogenase elevated PGE2^13^. Increased PGE2 level is thought to start vascular stimulation and cytokine-mediated tissue remodelling that eventually leads to hyperhidrosis, acro-osteolysis, periostosis, arthritis and pachyderma as seen in PDP patients^14^. The major diagnostic criteria for PDP includes pachyderma, periostosis and finger clubbing with minor criteria such as hyperhidrosis, arthralgia, gastric ulcer, cutis verticis gyrate, blepharoptosis, joint effusion, column-like legs, oedema, seborrhoea, acne and flushing^15^. Our patient met all three major criteria and was diagnosed with a complete form of PDP.

For the above-mentioned signs and symptoms, possible differentials would be acromegaly, psoriatic arthritis, thyroid acropachy, etc^4,16,17^. Acromegaly, contrary to PDP presents with larger bones in the skull, face and limbs, jaw prognathism and associated increase in insulin-like-growth factor-1 levels and positive oral glucose tolerance test^4^. In our case, a thorough clinical examination and negative acromegaly-related indicators helped us rule out this differential. Psoriatic arthritis such as psoriatic onycho-pachydermo-periostitis being limited to extremities with psoriatic nail changes and skin lesions, can also be excluded^16^. Thyroid acropachy manifests as periostosis and arthralgia with progressive exophthalmos, relatively symmetric swelling of the hands and feet, clubbing of the digits and pretibial myxoedema closely resembling PDP^17^. Clinical history and examination along with lab investigations suggested normal thyroid function in our case. Radiographic and clinical findings in our case were similar to a case-series from Tunisia that reported marked periostosis of the long bones and pachyderma in all six cases with athralgia and clubbing in five patients^18^. In conjunction with this, our radiographical picture also showed melorheostosis of the right and left fibula. Melorheostosis is any rare sclerosing periosteal proliferation affecting appendicular bones in a limited segmental form demonstrating the characteristic ‘dripping candle wax’ appearance^19^. As PGE2 plays a dominant role in disease pathogenesis, the management can be done symptomatically using NSAIDs, corticosteroids, or colchicine^7^. A case study of a 26-year-old man born to healthy non consanguineous parents with similar clinical symptoms taking etoricoxib (60 mg per day) was shown to have improvement within 1 week^8^. Study shows alleviation of articular symptoms, folliculitis and pachyderma by colchicine whereas pachyderma and cutis vetricis gyrate improved with Isotretinoine^20,21^. Likewise, another article showed greater yield with the combination of etoricoxib and aescin to relieve joint pain while decreasing the inflammation without compromising liver and renal function in a 1-year follow-up time^7^. Similar to the studies, our patient also responded to etoricoxib treatment for 7 months with noticeable improvement of clinical symptoms like profuse sweating, thickening of skin over the forehead and easy fatigability, etc. Currently, there is no record of symptoms recurrence as the patient is continuing his medications on a follow-up basis.

As our case report has the limitation of the unavailability of long-term follow-up data or the absence of a control group, this creates potential avenues for future research regarding larger clinical studies regarding its vague pathophysiology, inconclusive treatment and for warranting long-term use of etoricoxib. Thus, thorough clinical and laboratory investigations should be done for patients with hypertrophic osteoarthropathy to rule out other differentials and treatment with selective NSAIDs like etoricoxib can be recommended.

## Conclusion

A high index of suspicion is required to identify PDP owing to its rare incidence and similar presentation with acromegaly, psoriatic arthritis and thyroid acropachy. Considering the efficacy and safety, management with etoricoxib seems to be promising as it significantly reduced inflammatory reactions and relieved or retarded pachydermia progression. However, the long-term effects of etoricoxib on PDP and the recurrence of signs and symptoms months or years after its discontinuation requires further studies.

## Ethical approval

Institutional Review Committee of Kathmandu University School of Medical Sciences (KUSMS-IRC) has agreed for the exemption from ethical approval for our retrospective case report according to the requirements for exemption adhering to policies and guidelines of the committee as our report involves single de-identified patient’s data without any experimental impact on patient care.

## Consent

Written informed consent was obtained from the patient for publication of this case report and any accompanying images. A copy of the written consent is available for review by the Editor-in-Chief of this journal.

## Sources of funding

None.

## Author contribution

N.V., N.A., S.K., S.A., and U.P.: involved in literature review, manuscript writing, and proofreading; N.V.: corresponding author, involved in literature review, manuscript writing, and proofreading; N.V. and N.A.: involved in patient examination and care, literature review, manuscript writing, and proofreading; N.A.: involved in imaging interpretation and proofreading.

## Conflicts of interest disclosure

The authors declare that they have no financial conflict of interest with regard to the content of this report.

## Research registration unique identifying number (UIN)

1. Name of the registry: not applicable.
2. Unique identifying number or registration ID: not applicable.
3. Hyperlink to your specific registration (must be publicly accessible and will be checked): not applicable.

## Guarantor

Nirish Vaidya.

## Data availability statement

All supporting documents are submitted along with the case report.

## Provenance and peer review

Not commissioned, externally peer-reviewed.
